# Immune-mediated inflammatory diseases with chronic excess of serum interleukin-18

**DOI:** 10.3389/fimmu.2022.930141

**Published:** 2022-07-25

**Authors:** Hanae Miyazawa, Taizo Wada

**Affiliations:** Department of Pediatrics, School of Medicine, Institute of Medical, Pharmaceutical and Health Sciences, Kanazawa University, Kanazawa, Japan

**Keywords:** hemophagocytic lymphohistiocytosis, inflammasome, interleukin-18, macrophage activation syndrome, systemic autoinflammatory diseases

## Abstract

**Review**: Interleukin-18 (IL-18) is a proinflammatory cytokine that promotes various innate immune processes related to infection, inflammation, and autoimmunity. Patients with systemic juvenile idiopathic arthritis and adult-onset Still’s disease exhibit chronic excess of serum IL-18, which is associated with a high incidence of macrophage activation syndrome (MAS), although the mechanisms of IL-18 regulation in such diseases remain largely unknown. Similar elevation of serum IL-18 and susceptibility to MAS/hemophagocytic lymphohistiocytosis (HLH) have been reported in monogenic diseases such as X-linked inhibitor of apoptosis deficiency (i.e., X-linked lymphoproliferative syndrome type 2) and NLRC4-associated autoinflammatory disease. Recent advances in molecular and cellular biology allow the identification of other genetic defects such as defects in *CDC42*, *PSTPIP1*, and *WDR1* that result in high serum IL-18 levels and hyperinflammation. Among these diseases, chronic excess of serum IL-18 appears to be linked with severe hyperinflammation and/or predisposition to MAS/HLH. In this review, we focus on recent findings in inflammatory diseases associated with and probably attributable to chronic excess of serum IL-18 and describe the clinical and therapeutical relevance of understanding the pathology of this group of diseases.

## Introduction

**Figure 1:**
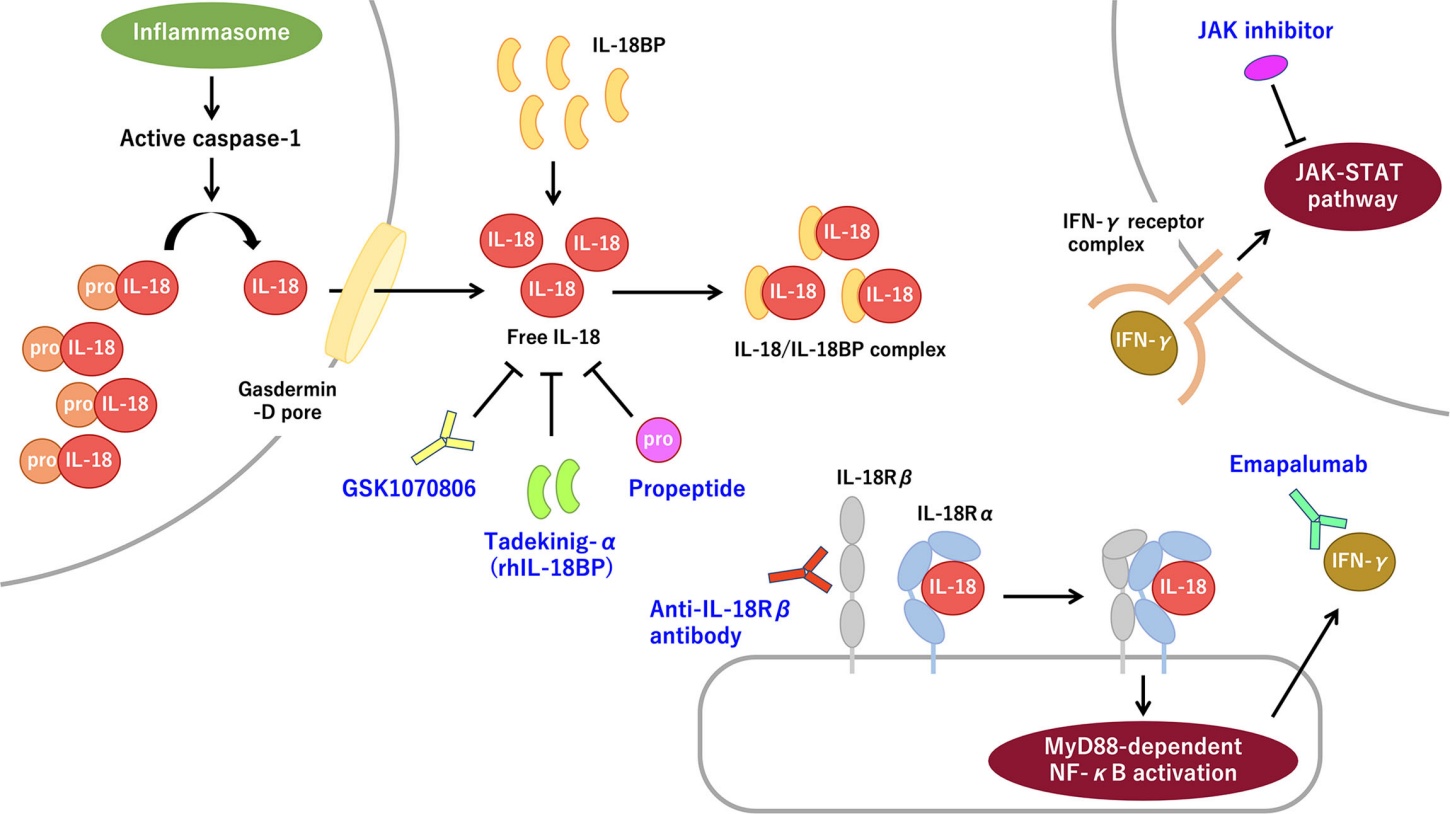
The IL-18 signaling pathway and promising molecular targeted therapies for IL-18 and IL-18-related proteins. Pro-IL-8 is constitutively expressed and accumulated intracellularly. Inflammasome-mediated caspase-1 activation induces pro-IL-18 cleavage. Mature IL-18 is released through the gasdermin-D pore and binds with low affinity to IL-18Rα, thereby recruiting IL-18Rβ. The IL-18/IL-18R complex induces MyD88-dependent NF-κB activation, which subsequently promotes IFN-γ secretion primarily from T-cells and NK cells. IFN-γ promotes strong proinflammatory signaling through the IFN-γ receptor-mediated JAK–STAT pathway. The extracellular IL-18 mostly exists as the inactive form of the IL-18/IL-18BP complex. Free IL-18, bioactive unbound form of IL-18, is virtually absent in normal and most pathological conditions. The rhIL-18BP tadekinig-α is the most well-recognized IL-18-targeting therapy. Humanized anti-IL-18 monoclonal antibody, called GSK1070806, and IL-18 propeptide can also inhibit IL-18 signaling. Anti-IL-18Rβ (i.e., IL-1R7) antibody can specifically block IL-18Rβ without interfering with anti-inflammatory IL-37 signaling. Inhibiting the IFN-γ signaling pathway by using the anti-IFN-γ antibody emapalumab or a JAK inhibitor, is a promising therapeutic strategy for IL-18-mediated systemic autoinflammatory diseases. IFN-γ, interferon-γ; IL, interleukin; IL-18BP, interleukin-18 binding protein; IL-18Rα/β, interleukin-18 receptor α/β chain; IL-1R7, interleukin-1 receptor 7; JAK, Janus kinase; MyD88, myeloid differentiation primary response 88; NF-κB, nuclear factor-κB; NK, natural killer; rhIL-18BP, recombinant human IL-18 binding protein; STAT, signal transducer and activator of transcription.

Interleukin (IL)-18 (IL-18) is a pleiotropic proinflammatory cytokine that belongs to the IL-1 family. Transcription of IL-18 precursor, pro-IL-18, can be induced after various stimulations including Toll-like receptor (TLR), which induces nuclear factor-κB (NF-κB) activation. In contrast to pro-IL-1β, pro-IL-18 is constitutively expressed and is ready to be processed and released on inflammatory stimuli (1, 2). Pro-IL-18 bears the N-terminal propeptide sequence, which is removed to create optimal N-terminus to exert bioactivity (3, 4). Multimolecular complexes of innate immune sensors, called inflammasomes, induce the autocatalytic activation of intracellular caspase-1 and promote pro-IL-18 processing. Active caspase-1 also induces inflammatory programmed cell death, called pyroptosis, thereby resulting in gasdermin-D cleavage, membrane pore formation, and the release of mature IL-1β and IL-18 (**Figure 1**). Several pathways also exist that mediate caspase-1-independent pro-IL-18 processing (2, 5). In humans, pro-IL-18 is primarily expressed in blood monocytes, macrophages, and dendritic cells, but it is also expressed in nonhematopoietic cells such as endothelial cells, keratinocytes, intestinal epithelial cells, and Kupffer cells (1).

With regard to the ligand–receptor interaction, mature IL-18 forms a low-affinity complex with the IL-18 receptor α chain (IL-18Rα; also called IL-1R5) and subsequently recruits the IL-18 receptor β chain (IL-18Rβ; also called IL-1R7), thereby forming a high-affinity complex, which induces myeloid differentiation primary response 88 (MyD88)-dependent NF-κB activation (**Figure 1**) (6, 7). IL-18 signaling contributes to innate and adaptive immune responses. IL-18 primarily induces interferon-γ (IFN-γ) from T-cells and natural killer (NK) cells in concert with IL-12 and IL-15. Furthermore, IL-18 directly upregulates cytotoxic activity of NK cells and cytotoxic T-cells. IL-18 also induces IL-17 secretion by γδ T-cells, which may have a role in autoimmune responses. In the absence of IL-12 and IL-15, IL-18 induces Th2 responses instead (1–3).

In terms of regulatory processes, IL-18 binding protein (IL-18BP) is the most well-recognized endogenous regulator of IL-18. Serum IL-18BP is constitutively expressed in a large amount and binds with IL-18 with high affinity; therefore, circulating IL-18 mostly exists as the inactive form of the IL-18/IL-18BP complex (**Figure 1**). Indeed, free IL-18, bioactive unbound form of IL-18, is virtually absent in normal and most pathological conditions, indicating IL-18 activity is strictly controlled. IL-18BP is induced by IFN-γ stimuli, which indicates a negative feedback loop in IL-18 signaling (2, 8). IL-37, another regulator of IL-18, is a cytokine in the IL-1 family, which is produced in the precursor form and cleaved into the mature active form. Extracellular IL-37 binds with IL-18Rα and recruits decoy receptor interleukin-1 receptor 8 (IL-1R8) to form a complex, thereby inducing anti-inflammatory signaling (9). Of note, higher levels of IL-18BP probably binds to IL-37, thereby resulting in the deprivation of its anti-inflammatory effects.

Previous studies (10) provide evidence suggesting a significant correlation between excess serum IL-18 and macrophage activation syndrome (MAS). MAS is a life-threatening condition that is characterized by fever, splenomegaly, and typical abnormalities in laboratory markers. An IL-18/IL-18BP imbalance-induced free IL-18 appears to have a central role in MAS development *via* promoting IFN-γ-mediated macrophage activation (1). MAS belongs to secondary hemophagocytic lymphohistiocytosis (sHLH), which arises in the setting of rheumatic diseases or systemic autoinflammatory diseases (SAIDs), which are mostly associated with systemic juvenile idiopathic arthritis (sJIA) and adult-onset Still’s disease (AOSD). In contrast to MAS or sHLH, primary hemophagocytic lymphohistiocytosis (pHLH) or familial hemophagocytic lymphohistiocytosis (fHLH) indicates hemophagocytic lymphohistiocytosis (HLH) which occurs in the context of the genetic background or genetic defects in lymphocyte cytotoxicity (10). Dramatic elevation of serum IL-18 has recently been reported in some monogenic SAIDs (1, 10). Among the disorders, chronic excess of serum IL-18 seems to be associated with life-threatening hyperinflammation and/or MAS/HLH predisposition. These observations highlight the importance of having a better understanding of IL-18 biology and the importance of an IL-18-targeting therapy approach. In this paper, we focus on the molecular and clinical aspects of SAIDs with chronic elevation of serum IL-18. In this review, we distinctively use “MAS” or “HLH”, based on the terms conventionally used in relevant fields.

## Immune-mediated inflammatory diseases with chronic excess of serum IL-18

### sJIA/AOSD

sJIA is a chronic childhood arthritis with the predominant features of systemic inflammation such as a daily spiking fever, evanescent rash, lymphadenopathy, hepatosplenomegaly, and serositis (11). AOSD is the adult counterpart of sJIA, based on their clinical similarities; however, the pathological identity has not been certified (12). A recent genome-wide association study (13) revealed distinctive genetic loci associated with sJIA, although a definitive genetic background and the etiology of sJIA/AOSD have not been established. Recognizing sJIA/AOSD as “pure” SAIDs is challenging because of the strong genetic association with the major histocompatibility complex class II locus (14).

sJIA/AOSD is distinguished from other rheumatic diseases by its prevalent association with MAS. MAS occurs in approximately 10%–20% of sJIA/AOSD patients and it subclinically develops in up to 50% of sJIA patients (12, 15). Tremendous increased levels of serum IL-18 are recognized as a notable feature of sJIA/AOSD. Accumulating evidence suggests that the serum levels of IL-18 are positively correlated with disease activity and the risk of MAS development (16–19). The serum concentration of IL-18 is approximately 10^5^ pg/mL in the MAS phase, 10^4^–10^5^ pg/mL in the active phase, and 10^3^–10^4^ pg/mL in the inactive phase [actual values of serum IL-18 in each disease phase is available in ref (18).]. Monitoring serum IL-18 levels is also useful for predicting the clinical course (20). Despite the swift improvement of other proinflammatory cytokine levels, serum IL-18 is persistently elevated in the inactive phase with only mild improvement (16, 18, 19). An intriguing finding is that elevated free IL-18 levels owing to the relatively low levels of serum IL-18BP is strongly associated with sJIA and the prominent elevation is specifically observed in the MAS phase (16). A similar propensity has also been demonstrated in AOSD (21). Thus, the bioactive unbound form of IL-18 has a role in MAS development as a driver of IFN-γ and IFN-γ-induced proteins (22, 23).

The genetic contribution to the pathogenesis of sJIA/AOSD and MAS has been speculated in some studies (15). Genetic variants in pHLH-associated genes are highly observed in sJIA-MAS, compared to sJIA without MAS (24, 25). No clear correlation exists between sJIA-MAS and monogenic defects in cytotoxicity-related genes, although these genetic variants seem to lower the threshold for MAS development (15). By contrast, accumulating evidence suggests that acquired NK cell dysfunction could be a primary contribution to MAS development in sJIA (26–31). Several studies (15, 26–29) have indeed demonstrated that sJIA NK cells exhibit cytotoxic dysfunction and reduced IL-18-driven IFN-γ production despite the fact that IL-18 canonically augments cytotoxicity and cytokine production by NK cells. Cytotoxic dysfunction may partially be caused by NK lymphopenia or decreased cytotoxic protein expression (15). Defective IFN-γ production against IL-18 stimuli is substantiated by the presence of high levels of serum IL-18 and relatively low levels of IL-18BP and chemokine (C-X-C motif) ligand 9 (CXCL9), both of which are induced by IFN-γ (16). This discrepancy reflects the hyporesponsiveness to IL-18 that is likely caused by functional exhaustion of NK cells (15, 28). At the time of MAS, massively induced total IL-18 seems to surpass the amount of IFN-γ-driven IL-18BP, which consequently provokes the elevation of free IL-18 (16).

Inflammatory lung disease (LD) has recently been recognized as an emergent feature of sJIA, which is clinically associated with refractory MAS, early-onset sJIA, adverse reactions to biologic therapy, and high serum IL-18 (32). The predominant pathology is endogenous lipoid pneumonia/pulmonary alveolar proteinosis (PAP) (32, 33). Cytokine and gene expression profiling in bronchoalveolar lavage fluid and lung tissue indicates IFN-γ-induced pulmonary inflammation (32). Another study (34), which used diagnostic screening for undifferentiated SAIDs with type-I IFN-response-gene score and cytokine profiling, identified IL-18-mediated PAP and recurrent MAS (IL-18PAP-MAS). In that study, one-half of the patients were already diagnosed with sJIA, whereas the remaining patients did not have this diagnosis. These patients had extremely high serum IL-18 and an elevated IL-18/CXCL9 ratio, which was comparable to NLR family caspase activation and recruitment domain (CARD)-containing protein 4 (NLRC4)-MAS. Owing to the lack of conclusive etiology and genetic background, whether LD lies within the spectrum of sJIA or partially shares clinical features is unclear.

Cytokine blockade therapy such as the use of IL-1 and IL-6 antagonists exhibits favorable effects on sJIA/AOSD (35). A phase II clinical trial of recombinant human IL-18BP (rhIL-18BP), called tadekinig-α, showed safety and early signs of efficacy in refractory AOSD (36). To date, a phase II clinical trial for evaluating the safety and efficacy of the anti-IFN-γ antibody emapalumab for MAS complicating sJIA/AOSD has been completed (clinical trial no.: NCT03311854; it has not been published, although preliminary data indicate a promising therapeutic effect). In this regard, Janus kinase (JAK)–signal transducer and activator of transcription (STAT) pathway, which is downstream of various cytokine signaling pathways, including interferons (IFNs), can be a therapeutic target for sJIA/AOSD and its complications MAS and LD (37).

### X-linked inhibitor of apoptosis deficiency

X-linked inhibitor of apoptosis (XIAP) deficiency, also called X-linked lymphoproliferative syndrome type 2, is a rare inborn error of immunity that is caused by loss-of-function mutations in *XIAP/BIRC4* gene encoding the XIAP protein (38, 39). XIAP is a highly conserved and ubiquitously expressed protein that belongs to the inhibitor of apoptosis (IAP) family of proteins and consists of five functional domains: three baculovirus IAP repeat (BIR) domains; a ubiquitin-associated (UBA) domain; and a C-terminal really interesting new gene (RING) domain, which has E3 ubiquitin ligase activity. The disease-causing mutations including nonsense, missense, and large or small deletions and insertions are distributed along the length of the gene, but intriguingly, missense mutations cluster in two hot spots that target either the BIR2 domain or the RING domain, highlighting the functional relevance of these two domains. There is no clear correlation between genotype and phenotype (38, 39). XIAP displays a pleiotropic function in cell survival, innate immunity, and inflammation. XIAP inhibits apoptosis by directly inhibiting caspase-3, caspase-7, and caspase-9 through interaction with the BIR domains (38, 39). XIAP is also required for intracellular pattern recognition receptor nucleotide-binding oligomerization domain 1 (NOD1)/NOD2 signaling and transmembrane Dectin-1 signaling, which recognize the degradation products of the bacterial cell wall and fungal β-glucan, respectively, and eventually leads to NF-κB and mitogen-activated protein kinase activation and pathogen clearance (38, 39). Recent findings provide an additional function of XIAP in autophagy (40, 41).

The clinical features of XIAP deficiency are characterized by susceptibility to Epstein–Barr virus (EBV) infection, recurrent HLH with/without EBV, splenomegaly, inflammatory bowel disease (IBD), and hypogammaglobulinemia. Unlike in fHLH and X-linked lymphoproliferative disease type 1, the cytotoxic function of NK cells and cytotoxic T-cells is not impaired (42, 43), which implies that a mechanism other than cytotoxic dysfunction could be involved in recurrent HLH in XIAP deficiency. In 2014, one study (44), which used serum cytokine profiling in 10 patients with HLH, revealed marked elevation of serum IL-18 (**Table 1**). Of note, the serum IL-18 concentration was persistently elevated after recovery from HLH. These findings suggest that massive and chronic elevation of serum IL-18 may contribute to HLH susceptibility in XIAP deficiency. Previous studies (39, 63–67) have unraveled the function of XIAP in regulating the activation of nucleotide-binding oligomerization domain (NOD)-like receptor family pyrin domain containing 3 (NLRP3) inflammasome. First described was the ripoptosome pathway. On tumor necrosis factor receptor 1 (TNFR1) and TLR stimulation, the loss of IAPs, particularly XIAP, stimulates the formation and activation of ripoptosome (which consists of receptor-interacting protein kinase 1 [RIPK1], FAS-associated protein with death domain [FADD], and caspase-8), and promotes RIPK3-dependent NLRP3 activation and cell death (63). In this pathway, XIAP appears to control RIPK1 function by regulating its ubiquitination status. Activated caspase-8 can also mediate pro-IL-1β cleavage in an NLRP3-caspase-1-independent manner (65). If ripoptosome-induced caspase-8 activation is insufficient, then RIPK1 associates with RIPK3, thereby promoting the phosphorylation of mixed-lineage kinase domain-like by RIPK3 and eventually leading to NLRP3 activation and necroptosis (64). XIAP is also involved in the TNFR2 signaling pathway (66, 67). TLR-MyD88-induced TNF/TNFR2 signaling promotes the proteasomal degradation of the cellular IAP-1 (cIAP1)/TNF receptor-associated factor 2 complex. In the context of XIAP loss, TNFR2-induced cIAP1 degradation promotes TLR-RIPK3-caspase-8-induced NLRP3 activation (66). A more recent study (67) reports that TNFR2-induced NLRP3 activation occurs *via* TNF/TNFR1 signaling in the absence of XIAP, which is mediated by RIPK1 kinase activity and reactive oxygen species (ROS) production. Most of these findings are obtained from studies using mouse myeloid cells (63–67); however, these findings indicate that XIAP is an essential regulator of NLRP3 inflammasome activation.

**Table 1:** Immune-mediated inflammatory diseases with massive and chronic elevation of serum IL-18.

Disease	Gene	Inheritance	Serum IL-18(pg/mL)	Clinical findings	Reference
sJIA/AOSD	Polygenic	Non-hereditary	10^3^–10^5^	MAS, fever, rash, serositis, hepatosplenomegaly	(11, 18)
XIAP deficiency	*XIAP*	XR	10^3^–10^5^	Susceptibility to EBV infection, HLH, IBD, splenomegaly, hypogammaglobulinemia	(16, 44)
NLRC4-AID	*NLRC4*	AD	10^3^–10^5^	MAS, severe enterocolitis	(16, 45–52)
CDC42 C-terminal disease	*CDC42*	AD	10^4^	Pancytopenia, HLH, rash, hepatosplenomegaly, myelofibrosis/proliferation	(53–57)
PAID	*PSTPIP1*	AD	10^3^–10^4^	Sterile pyogenic arthritis, pyoderma gangrenosum, cystic acne	(58, 59)
WDR1 deficiency	*WDR1*	AR	10^3^	Recurrent stomatitis, thrombocytopenia, neutrophil abnormalities in migration and morphology, impaired adaptive and innate immunity, autoinflammation	(60–62)

AD, autosomal dominant; AIFEC, autoinflammation with infantile enterocolitis; AR, autosomal recessive; CDC42, cell division cycle 42; EBV, Epstein-Barr virus; HLH, hemophagocytic lymphohistiocytosis; IBD, inflammatory bowel disease; MAS, macrophage activation syndrome; NLRC4-AID, NLR family caspase activation and recruitment domain-containing protein 4-associated autoinflammatory disease; PAID, PSTPIP1-associated inflammatory diseases; PSTPIP1, proline-serine-threonine phosphatase-interacting protein 1; sJIA/AOSD, systemic juvenile idiopathic arthritis/adult-onset Still’s disease; WDR1, WD repeat-containing domain 1; XIAP, X-linked inhibitor of apoptosis; XR, X-linked recessive.

Why XIAP loss-induced NLRP3 hyperactivation induces excessive and chronic elevation of serum IL-18 is unresolved. Patients with cryopyrin-associated periodic syndrome, which is caused by NLRP3 gain-of-function mutations, are less likely to have recurrent MAS. Considering that XIAP is involved in TNFR1/TLR/TNFR2 signaling, XIAP loss-induced comprehensive activation of ripoptosome/necrosome pathways and/or NLRP3-independent pro-IL-18 processing may contribute to the massive and persistent production of mature IL-18. In addition, in TNFR2 signaling, the inhibition of RIPK1 kinase activity and ROS production significantly suppresses the release of IL-1β, but the release of IL-18 is not reduced to the baseline level (67). This finding may imply the possibility that distinct regulation systems, apart from that of IL-1β, and/or other inflammasome involvement contribute to hyper-IL-18 in the context of XIAP loss.

The curative therapy for XIAP deficiency is allogenic hematopoietic stem cell transplantation (HSCT), which should be considered for uncontrollable HLH or severe refractory IBD (39). To achieve a favorable outcome, patients should be treated with a reduced intensity conditioning regimen and HLH should be in remission before HSCT (68). Recurrent HLH is characteristic of XIAP deficiency and can occur without EBV infection (39). The existence of subclinical HLH has been substantiated by the finding of histopathological hemophagocytosis in the resected spleen (69). Recent report described the anti-inflammatory effect of rhIL-18BP in a patient with XIAP deficiency (70). IL-18-targeting therapy is expected to improve HLH susceptibility and may serve as a bridge to HSCT.

### NLRC4-associated autoinflammatory disease

NLRC4 is a cytosolic protein that forms an inflammasome in response to bacterial ligands, flagellin, and the type III secretion system. The NLRC4 inflammasome mediates the cleavage and activation of caspase-1, which results in the production of mature IL-1β and IL-18 (71, 72). Active caspase-1 also proteolytically activates gasdermin-D and induces pyroptosis. In the latter process, the adaptor protein apoptosis-associated speck-like protein containing a CARD (ASC) is not absolutely required (45). In humans, NLRC4 is expressed in the myeloid lineage, including circulating monocytes and neutrophils, and it is expressed in nonhematopoietic cells such as intestinal epithelial cell. Thus, it has important roles in host defense and autoinflammation (16, 46, 71, 72).

A monomeric NLRC4 consists of an N-terminal CARD, a C-terminal leucin-rich repeat (LRR), and a central NOD, which contains four domains: nucleotide-binding domain (NBD), helical domain 1 (HD1), winged helix domain (WHD), and helical domain 2 (HD2). Within the NOD, HD1, WHD, and HD2 form an ADP-binding pocket and stabilize NLRC4 autoinhibitory conformation. On LRR detection of the ligand-bound NLR family of apoptosis inhibitory protein (NAIP), which is a ligand sensor protein, NOD conformation changes to the active form, which promotes NLRC4 oligomerization and inflammasome assembly (71, 72). The intermolecular LRR–LRR interface is also important for NLRC4 oligomerization (45).

An NLRC4 gain-of-function mutation leads to the spontaneous activation of NLRC4 inflammasome and causes autoinflammation. To date, 13 pathogenic variants have been reported (45–52, 73–80). Most of them are located around the ADP-binding site, which abrogates nucleotide-binding autoinhibitory conformation. Recent independent studies have revealed three individual mutations in the LRR domain: p.W655C, p.Q657L, and 93bp in-frame deletion within exon 5, which likely affect the LRR–LRR oligomerization interface and induce spontaneous inflammasome activation (45, 51, 78). The origins of these pathogenic variants are inherited, *de novo*, or through somatic mosaicism. A recent study (79) described late-onset NLRC4-AID with low frequency somatic mosaicism (2%–4%).

NLRC4-AID represents a wide-spectrum of clinical phenotypes. NLRC4-MAS/autoinflammation with infantile enterocolitis (AIFEC) is the most severe and life-threatening form of the disease. By contrast, some cases involve a milder phenotype, which are diagnosed as familial cold autoinflammatory syndrome 4 (FCAS4) or neonatal-onset multisystem inflammatory disease (NOMID) (72). An argument initially was whether NBD and HD1 mutations were likely associated with NLRC4-MAS/AIFEC and the NOMID-phenotype, and whether WHD mutations were associated with the FCAS phenotype (72). However, in recent familial cases of the FCAS phenotype, patients harbored the novel pathogenic variant in the NBD domain (80), which indicated that a genotype–phenotype correlation is not as clear as might have been expected. Serum IL-18 is markedly and chronically elevated in NLRC4-AID. However, NLRC4-MAS/AIFEC exhibits more elevated serum IL-18 than does FCAS4 or NOMID (10^3^–10^5^ *vs*. 10^3^ pg/mL), thereby demonstrating that the levels of serum IL-18 are likely correlated with disease severity (45–52, 75, 76, 80) (**Table 1**).

Weiss *et al.* (16) reported that systemic IL-18 elevation is inflammasome-dependent and entirely derived from intestinal epithelia in NLRC4-MAS mice. Therefore, HSCT alone may not be a curative treatment option. A remarkable finding is that spontaneous clinical remission has been reported in patients with NLRC4-MAS/AIFEC after the first year of life, which may be associated with the maturation of the gut microflora (47, 50).

NLRC4-MAS/AIFEC unfortunately has a high mortality rate because of its aggressive and rapid progression. However, to date, an NLRC4-specific inhibitor has not been identified (71). The therapeutic effect of IL-1-blocking strategy varies among each case and the elevation of serum IL-18 persists, even after initiating IL-1-blocking therapy, which indicates that IL-18 is a therapeutic target (48, 50, 52, 75). The rhIL-18BP tadekinig-α is indeed effective for clinical and serological remission, which emphasizes the pathogenic role of IL-18 in NLRC4-MAS/AIFEC (16, 49). In one report (74), the anti-IFN-γ antibody emapalumab was also an efficacious treatment in one patient with NLRC4-MAS. A recent study (46) revealed the efficacy of the mTOR inhibitor rapamycin, which markedly decreases serum IL-18. Sasaki *et al.* (81) described the distinct role of IL-1β and IL-18 among tissues with NLRC4-induced autoinflammation. This finding suggests that cytokine-blocking therapy should be utilized by concerning the site of tissue inflammation.

### Cell division cycle 42 C-terminal disease

Cell division cycle 42 (CDC42) is an intracellular member of the Ras homology (Rho) GTPases, which control cell polarity by regulating the assembly of actin cytoskeleton structures. From an immunological point of view, CDC42 regulates multiple cellular processes such as migration, immunological synapse formation, and polarized cytokine secretion. CDC42 also has a critical role in cell proliferation and hematopoiesis (82–84). CDC42 exhibits reciprocal exchange between the GTP-bound active form and the GDP-bound inactive form. This process is mediated by guanine nucleotide exchange factors and GTPase-activating proteins. Rho GDP-dissociation inhibitors (RhoGDIs), another regulator of Rho GTPases, sequester CDC42 in cytosol away from membranes and modulate reversible cytosol-membrane cycle of CDC42 through interaction with the prenylated form of CDC42 (82, 83). The intracellular localization and function of CDC42 is regulated by the posttranslational geranyl-geranylation at Cys188 and the binding affinity for RhoGDIs (82, 83, 85).

Previous studies (86–91) demonstrate that pathogenic missense mutations in CDC42 cause a wide-spectrum of clinical features such as neurodevelopmental abnormality, dysmorphism, and hematologic and immunological dysfunction. A novel group of *CDC42* missense variants in the C-terminal region have recently emerged: p.R186C, p.C188Y, and p.*192C*24, which dominantly manifest as autoinflammation and hematologic abnormalities (53–57). This new phenotype is characterized by neonatal-onset cytopenia with dyshematopoiesis, autoinflammation, rash, and HLH (NOCARH) syndrome. Patients exhibit a strikingly dramatic excess of serum IL-18 (i.e., 10^4^ pg/mL) (53, 54) (**Table 1**). The plasma levels of IFN-γ and CXCL9 are correlated with HLH flares, whereas serum IL-18 levels are constitutively elevated and persist after initiating IL-1-targeting therapy (53, 54). These findings suggest that spontaneous inflammasome activation and IL-18 overproduction could be associated with susceptibility to HLH.

The findings of subsequent studies (53, 57, 82) imply that NF-κB-dependent proinflammatory cytokine production and/or impaired NK cell cytotoxicity may contribute to autoinflammation in CDC42 C-terminal disease. Findings of a recent investigation alternatively suggest that aberrant-palmitoylation-induced Golgi trapping of CDC42 can induce pyrin inflammasome overactivation (85). The process is not associated with modified GTPase activity or binding affinity for RhoGDIs. Aberrant Golgi distribution occurs in CDC42^R186C^ and CDC42^*192C*24^, which is correlated with the levels of IL-1β production; however, CDC42^C188Y^, which presumably lacks posttranslational geranyl-geranylation, predominantly exhibits cytoplasmic and nuclear distribution and does not show IL-1β overproduction. The discordance between the *in vitro* findings and the clinical inflammatory phenotypes shared by C-terminal variants remains unclear. Furthermore, the molecular mechanism of pyrin inflammasome overactivation induced by subcellular mislocalization of mutated CDC42 has not been provided.

CDC42 C-terminal disease exhibits aggressive and life-threatening inflammation without appropriate immunosuppressive therapy. However, the clinical course seems to be heterogeneous, depending on genotype. Recent evidence suggests that p.R186C, rather than p.C188Y and p.*192C*24, is specifically correlated with neonatal-onset severe pancytopenia and autoinflammation/HLH (92). Cytopenia in p.C188Y and p.*192C*24 is IL-1 blocker-sensitive, which indicates inflammation-induced cytopenia, although autoinflammation/HLH in p.R186C generally exhibits hyporesponsiveness to IL-1-targeting therapy. These findings suggest that the inflammatory response in p.R186C may depend more strongly on IL-18 than on p.C188Y and p.*192C*24. Of note, in one report (53), anti-IFN-γ antibody and subsequent HSCT was curative for one patient with p.R186C. The existence of chronic elevation of serum IL-18, even after clinical responsiveness to anti-IL-1 therapy, suggests that IL-18 is a promising therapeutic target for CDC42 C-terminal disease (54).

### Pyogenic sterile arthritis, pyoderma gangrenosum, and acne syndrome and its subtypes with *PSTPIP1* pathogenic mutations (i.e., PSTPIP1-associated inflammatory diseases)

Pyogenic sterile arthritis, pyoderma gangrenosum, and acne (PAPA) syndrome is a hereditary autosomal dominant SAID, which is characterized by early-onset destructive sterile pyogenic arthritis with variable skin manifestations such as pyoderma gangrenosum (PG) and cystic acne in adolescence and beyond (93). PAPA syndrome is caused by gain-of-function mutations in the proline-serine-threonine phosphatase-interacting protein 1 (*PSTPIP1*) gene encoding the cytoskeleton-associated adaptor protein PSTPIP1, which is expressed predominantly in hematopoietic cells (58, 94). PSTPIP1 consists of N-terminal Fes/CIP4 homology-Bin-Amphiphysin-Rvs (F-BAR) domain, which contain the Fes/CIP4 homology domain and coiled-coil (CC) domain; rich in proline, glutamic acid, serine, and threonine (PEST) residues domain; and the C-terminal Src-homology 3 (SH3) domain (93, 95). PSTPIP1 interacts with various types of proteins, including pyrin, PEST-type protein tyrosine phosphatase (PTP-PEST), and Wiskott–Aldrich syndrome protein (WASp). The representative PAPA-associated mutations p.E250Q and p.A230T are in the CC domain and impair the interaction with PTP-PEST, which promotes hyperphosphorylation of PSTPIP1. This process increases the affinity of PSTPIP1 to pyrin, thereby leading to pyrin inflammasome activation and IL-1β production (94–97). By contrast, other disease-causing mutations in the CC domain—p.E250K and p.E257K—induce significant charge switch of the PSTPIP1 surface relative to p.E250Q, which further enhances the binding of PSTPIP1 to pyrin. These mutations cause more severe and early-onset inflammatory disease, PSTPIP1-associated myeloid-related proteinemia inflammatory (PAMI) syndrome, which is characterized by cutaneous inflammation, hepatosplenomegaly, failure to thrive, pancytopenia, and joint involvement (98).

Several reports demonstrate the primary role of PSTPIPT1 in cytoskeletal organization. PSTPIP1 directly or indirectly associates with microtubules to organize the PSTPIP1 filament network, the dynamics of which are modulated by pyrin (95). In addition, PSTPIP1 also interacts with the actin-regulating protein WASp through the SH3 domain (99). PSTPIP1 serves as a scaffold protein of a complex with WASp and PTP-PEST, thereby allowing PTP-PEST to dephosphorylate WASp and to regulate its function (99, 100). In stimulated cells, PSTPIP1-pyrin colocalizes with actin at the leading edge of the cells and regulates phagocyte migration and invasion (101, 102). In the SH3 domain, p.R405C causes constitutive activation of WASp, which induces filopodia formation and matrix degradation. This mutation is associated with severe skin manifestations without arthritis, pancytopenia, and organomegaly, which is called the PG, acne, suppurative hidradenitis (PASH) syndrome (103). As described previously, inflammatory disease caused by *PSTPIP1* pathogenic mutations encompasses several subgroups with a distinctive phenotype and genotype; therefore, these related diseases are collectively described as PSTPIP1-associated inflammatory diseases (PAID) (93).

In 2017, hyper-IL-18 in PAID was first detected in one report (58). The patient presented with facial acne, splenomegaly, PG, and pancytopenia (i.e., a PAMI-like phenotype), but the patient lacked a genetic evaluation. PG and pancytopenia were well controlled after starting cyclosporin; however, splenomegaly and acne did not have a remarkable improvement. Elevated serum IL-18 was notably retained after the improvement of PG (before starting cyclosporin: 87,900 pg/mL; after the improvement of PG: 85,500 pg/mL). In 2021, massive and chronic elevation of serum IL-18 in patients with PAID was described (59) (**Table 1**). The levels of serum IL-18 in these patients were as high as those of patients with NLRC4-MAS and higher than those of patients with familial Mediterranean fever. In addition, dramatically high levels of serum IL-18 constitutively exist and are not associated with disease activity and the level of C-reactive protein. It is important to note that PAID is less likely to be affected with recurrent MAS/HLH, nevertheless free IL-18 is detected at all time points. An intriguing finding is that patients with the PAPA- and PAMI-associated genotypes have persistent hyper-IL-18 levels, whereas the levels of serum IL-18 in patients with the PAPA-like condition, including patients with a PASH-associated mutation or no pathogenic variants, are largely in the normal range. From a molecular basis, IL-18 overproduction in PAPA and PAMI syndrome may predominantly depend on pyrin inflammasome activation. However, a recent study (104) revealed NLRP3 involvement in PAPA and PAMI syndrome, which suggests that IL-18 overproduction in PAID may be generated against a background of more complicated mechanisms. Anti-inflammatory therapy with steroids, cyclosporine, and anti-TNFα has been proposed for PAID, although a definitive therapeutic approach has not been established. The fact that anti-IL-1 therapy has favorable therapeutic effects on PAPA and PAMI syndrome reflects the pathogenic relevance of inflammasome activation; however, consistent effectiveness was not observed among the group of patients (98, 104). To date, no study has evaluated the efficacy of IL-18-targeting therapy for PAID.

### WD repeat-containing domain 1 deficiency

WD repeat-containing domain 1 (WDR1; also called actin interacting protein 1), which is encoded by the *WDR1* gene, is an actin regulatory protein that enhances the ability of cofilin to sever and depolymerize F-actin. This process is required for the remodeling of the actin filament (60). Biallelic loss-of-function mutations in the *WDR1* gene impair innate and adaptive immunity, thereby leading to various phenotypes (60–62). Previous studies (60–62, 105) have reported 12 patients in 7 families who were characterized by severe recurrent stomatitis, poor wound healing, immunodeficiency, and autoinflammation. Laboratory findings indicate impaired migration and morphological abnormality in neutrophils, thrombocytopenia due to dysfunctional platelet shedding, and developmental and functional impairment of T-cells and B-cells. B-cell dysfunction appears more severe than the T-cell compartment (62). WDR1 comprises 14 WD40 repeats, which form dual β-strand-based propellers with seven blades each (61). Pathogenic mutations are not strictly localized and a distinct genotype–phenotype correlation does not exist (60–62).

In 2007, Kile *et al.* (105) established mice homozygous for a hypomorphic allele of *WDR1* (*WDR1^rd/rd^*). Severe loss-of-function at the *WDR1* locus caused embryonic lethality, whereas *WDR1^rd/rd^* mice developed spontaneous autoinflammation and macrothrombocytopenia. In 2015, Kim *et al.* (106) determined that autoinflammation in *WDR1^rd/rd^* is driven by IL-18 rather than by IL-1β. In this regard, *WDR1^rd/rd^* monocytes, rather than dendritic cells (DCs), macrophages, and neutrophils, have a central role in the production of excess IL-18, which is induced by aberrant actin depolymerization-driven pyrin inflammasome activation. Predominant contribution of IL-18 but not IL-1β has also been reported in human WDR1 deficiency (61). Two affected siblings from consanguineous parents experienced periodic fever, oral and perianal inflammation, and immunodeficiency, and had an elevated serum IL-18 level (**Table 1**), which was higher than the level in sJIA. One patient had persistent elevation of the serum IL-18 level, but the other patient had fluctuations in the serum IL-18 levels that were associated with the levels of acute phase reactants. The levels of serum IL-18BP were not elevated. Monocyte-derived DCs (moDCs) produced higher levels of serum IL-18 in one patient, compared to the levels in healthy controls, at baseline and after lipopolysaccharide treatment. However, the level of IL-1β was comparable to that of healthy controls. The patient-derived moDCs also had higher levels of polymerized F-actin and abnormal intracellular aggregates of WDR1. Furthermore, HEK293T cells transfected with pyrin and mutated *WDR1* had pyrin colocalized with mutated WDR1 aggregates. These findings suggest that the perturbation of the actin cytoskeleton, which is caused by dysfunctional WDR1, may induce autoinflammation through pyrin inflammasome activation. The pathogenic contribution of IL-18 is speculated by the fact that IL-1-targeting therapy showed a partial response in one patient who was treated with anakinra (61). However, not every patient develops autoinflammation or exhibits elevated serum IL-18 (60–62). In contrast to NLRC4-AID, the therapeutic effect of HSCT on the autoinflammatory phenotype highlights the central role of hematopoietic cells in producing excess IL-18 (61). The detailed mechanism of how actin dysregulation induces spontaneous pyrin inflammasome activation remains to be solved.

### Therapeutic strategies for SAIDs with chronic excess of serum IL-18

IL-18 and the downstream molecules of IL-18 signaling are promising therapeutic targets. The rhIL-18BP tadekinig-α has been proven as a safe and efficient treatment for refractory AOSD (36). Anti-inflammatory effects of rhIL-18BP are also observed in patients with NLRC4-MAS and XIAP deficiency (49, 70). The therapeutic efficacy and safety of rhIL-18BP continues to study in clinical trials for these diseases (clinical trial nos.: NCT03113760 and NCT03512314). In addition, rhIL-18BP has a favorable effect on refractory sJIA (107). The humanized anti-IL-18 monoclonal antibody GSK1070806 may be of benefit in suppressing IL-18-mediated autoinflammation (1). Anti-IL-18Rβ (i.e., IL-1R7) antibody specifically blocks IL-18 signaling without interfering with the anti-inflammatory IL-37 pathway (108). IL-18 propeptide interacts with mature cleaved IL-18 recapitulating the intramolecular inactivation form of pro-IL-18 (4).

Interfering with IL-18-evoked IFN-γ signaling is a promising strategy. The clinical trial of the anti-IFN-γ antibody emapalumab had a potential therapeutic effect for MAS complicating sJIA/AOSD (clinical trial no.: NCT03311854). Anti-IFN-γ antibody is also effective for some patients with NLRC4-MAS and CDC42 C-terminal disease (53, 74). Accumulating evidence suggests that the JAK-STAT pathway is a prospective therapeutic target for MAS/HLH (10, 109). Clinical trials of the JAK inhibitors baricitinib and tofacitinib for sJIA are ongoing (clinical trial nos.: NCT04088396 and NCT03000439).

To date, HSCT is an ultimate therapy for XIAP deficiency, CDC42 C-terminal disease, or WDR1 deficiency refractory to conventional treatment (39, 53, 61). Strategies for blocking IL-18 signaling pathway are summarized in **Figure 1**.

## Discussion

Technological evolutions in genetic analysis have allowed the successive identification of novel monogenic SAIDs. Pathological classification of monogenic SAIDs has been modified in accordance with newly unraveled molecular pathways. Among these, inflammasomopathies, that is, IL-1-family-mediated conditions are a major category of SAIDs (110–112).

**Figure 2:**
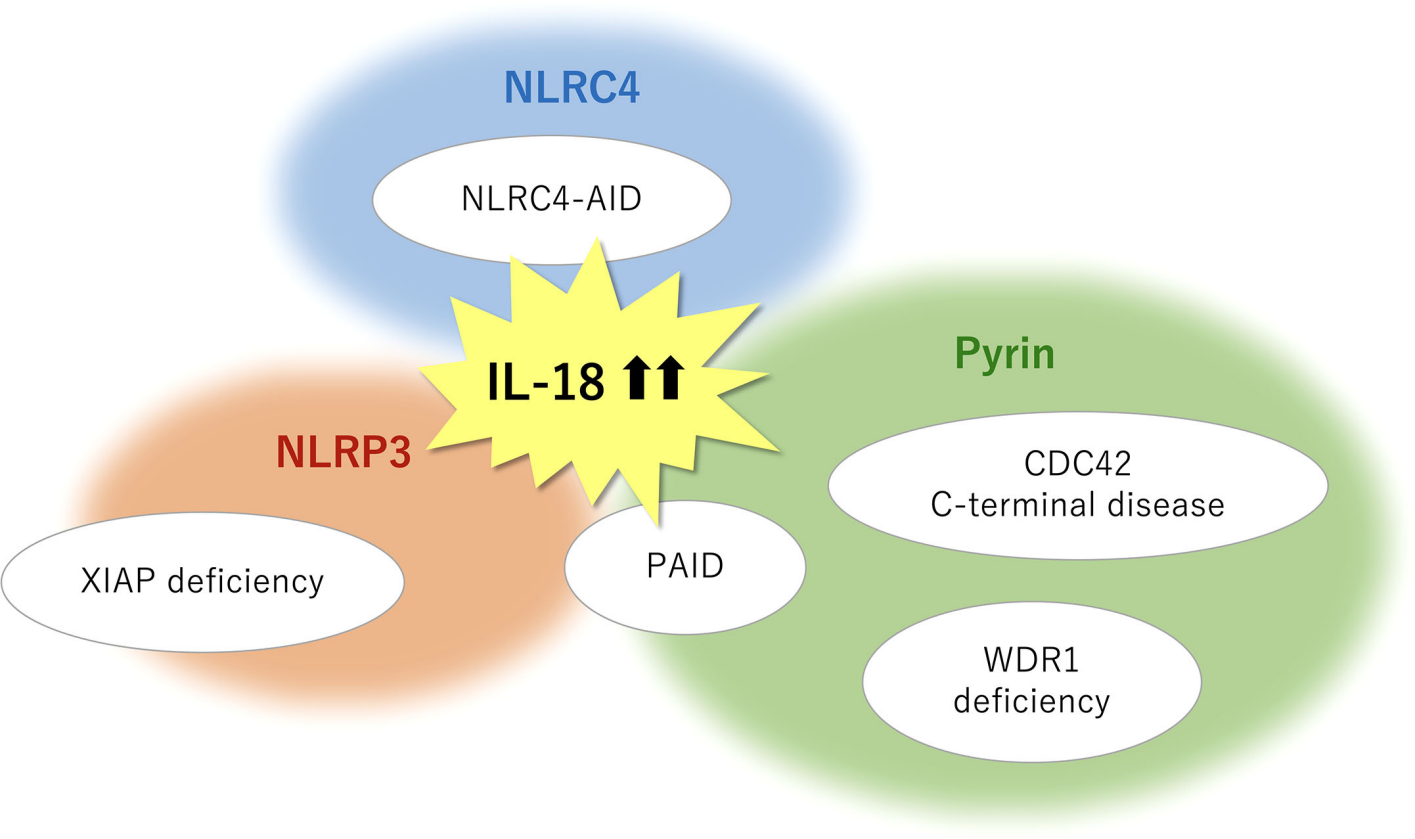
Monogenic SAIDs with chronic elevation of serum IL-18 and the relevant inflammasomes. NLRC4-AID is caused by gain-of-function mutations in the *NLRC4* gene and is characterized by a high incidence of macrophage activation syndrome. The chronic nature of IL-18 elevation observed in XIAP deficiency primarily depends on the NLRP3 inflammasome, whereas an inflammasome-independent pathway may in part have a role in mature IL-18 overproduction. Pyrin inflammasome is associated with IL-18-mediated autoinflammation in actin remodeling defects such as CDC42 C-terminal disease, PSTPIP1-associated inflammatory diseases (PAID), and WDR1 deficiency. Pyrin may serve as a sensor for actin-cytoskeleton dynamics. NLRP3 involvement is also observed in several subtypes of PAID such as PAPA syndrome and PAMI syndrome. SAIDs, systemic autoinflammatory diseases; CDC42, cell division cycle 42; IL, interleukin; NLRC4-AID, NLR family caspase activation and recruitment domain-containing protein 4-associated autoinflammatory disease; NLRP3, NLR family pyrin domain containing 3; PAMI, PSTPIP1-associated myeloid-related proteinemia inflammatory; PAPA, pyogenic sterile arthritis, pyoderma gangrenosum, and acne; PSTPIP1, proline-serine-threonine phosphatase-interacting protein 1; WDR1, WD repeat-containing domain 1; XIAP, X-linked inhibitor of apoptosis.

High concentrations of circulating total IL-18 have been reported in several clinical conditions including IBD, sepsis, type 2 diabetes, and atherosclerosis and myocardial infarction (2); however, it appears to date, that dramatic and often chronic elevation of serum IL-18 (> 10^3^–10^4^ pg/ml) are only linked to genetic diseases affecting its release or maybe production and a certain rheumatic disease (i.e., sJIA/AOSD). To exert a biological effect, IL-18 is produced in a precursor form, processed by caspase-mediated cleavage, and released into the extracellular milieu. Pro-IL-18 transcription can be induced by NF-κB activation following TLR stimulation. In contrast to pro-IL-1β, pro-IL-18 is constitutively expressed and accumulated intracellularly (1). Genetic defects associated with IL-18-related SAIDs may affect the induction of inflammasomes and cytokines expression (i.e., priming); however, this point is not clearly understood. XIAP loss may possibly influence on priming, because it is well known as an inhibitory factor of inflammatory cell death necroptosis (15). The cleaved and released process primarily depends on inflammasomes (**Figure 1**). Among the IL-18-related SAIDs, the relevant inflammasomes have been tentatively identified (**Figure 2**); however, why chronic hyper-IL-18 is restricted to certain diseases is not understood. A possibility is that the existence of chronic hyper-IL-18 in some SAIDs has not been recognized because of poor evaluation for the serum cytokine profile. Tissue- or lineage-specific contribution has been addressed in some diseases (e.g., epithelial specific inflammasome in NLRC4-MAS and dominant role of monocytes in WDR1 deficiency) (16, 106). The predominant expression of IL-18 rather than IL-1β has appeared in murine and human epithelia such as the gut and skin. The expression of NLRC4 is similarly higher than that of NLRP3 and pyrin in the murine intestinal epithelia. Mice with mutated NLRC4 have increased turnover of intestinal epithelia without histological abnormalities, which likely contribute to IL-18 overproduction (16). The basal expression of IL-18 and pyrin is also relatively prominent in human and murine monocytes (16, 106). Thus, the chronic elevation of IL-18 may in part depend on the expression levels of the relevant inflammasomes and cytokines in certain cell types.

Pathogenic variants in genes responsible for cytoskeletal organization can induce various types of immune dysregulation (113). Actin remodeling defect (i.e., actinopathies) is a major group of SAIDs. CDC42 C-terminal disease, PAID, and WDR1 deficiency are recognized as actin-related SAIDs with excess serum IL-18. Based on the close relationship between pyrin and actin-related molecules, pyrin may function as a sensor for the modification of the actin cytoskeleton, whereas the precise mechanism in this process remains unclear (61, 106, 114). Further information about the serum cytokine profile will identify other actin-related SAIDs with chronic hyper-IL-18, which is expected to provide insights into the molecular mechanisms underlying actin modification-induced pyrin inflammasome overactivation.

As indicated in XIAP deficiency and PAID, the molecular basis of chronic elevation of serum IL-18 is more intricate than previously thought and involves complicated interactions between multiple inflammasomes and cell death regulators. A new concept of inflammatory cell death, called PANoptosis, has recently emerged. PANoptosis is regulated by a molecular complex involving effector molecules from pyroptotic, apoptotic, and necroptotic (PAN) pathways, known as a PANoptosome (115). At least NLRP3, pyrin, and absent in melanoma 2 (AIM2, a sensor of double-strand DNA) are members of the PANoptosome (115, 116).

The pathological importance of IL-18 has been largely understood from findings in sJIA/AOSD and their most severe complication MAS. Indeed, the levels of serum IL-18 are positively correlated with disease activity and the risk of MAS development (17–19). A remarkable finding is that serum levels of IL-18BP are also elevated in sJIA/AOSD patients, but no significant difference exists in the levels of serum IL-18BP between active and inactive phase (21, 23). In addition, elevated levels of detectable free IL-18 due to relatively low levels of serum IL-18BP is prominently observed during MAS phase (16). Consistently, IL-18/IL-18BP imbalance has already been reported in sHLH in 2005 by Mazodier, et al. (117). These findings suggest that IL-18/IL-18BP imbalance is a significant factor for the development of MAS. The protective role of IL-18BP in MAS is also observed in the study using CpG-treated *IL-18BP^-/-^* mice (8). The question is why this phenomenon occurs despite the fact that IL-18BP expression is mostly IFN-γ-dependent, which indicates a negative feedback loop of IL-18/IFN-γ axis (118). The disruptive regulation of IL-18 bioactivity may in part reflect NK cell hyporesponsiveness toward IL-18. NK cells from sJIA patients present reduced IFN-γ production against IL-18 stimuli, which at least partially results from a reduced phosphorylation downstream of IL-18 receptor β (23, 26). Of note, IL-18/IL-18BP imbalance seems to be correlated with the risk of MAS, as seen in sJIA-MAS and NLRC4-MAS (16, 34); however, several subtypes of PAID exhibiting IL-18/IL-18BP imbalance are less likely to be associated with high incidence of MAS (59). The reason for these conflicting observations is still unclear, but higher levels of serum IL-18 beyond the threshold, a second factor that triggers hyperinflammation, and/or other regulatory or anti-inflammatory cytokines, such as IL-37 and IL-10, are probably indispensable for defining MAS/HLH predisposition. These observations point out that the pathogenesis of MAS/HLH can be multifactorial, but not only determined by IL-18 dysregulation. Intriguingly, one report demonstrates a protective role of intrahepatic IL-18BP for liver-specific inflammation in a patient carrying homozygous loss-of-function mutation in *IL-18BP* who succumbed to fulminant hepatitis due to hepatitis A virus (119). This finding indicates that exogenous factors and/or tissue specificity could also affect the clinical phenotype in the genetically determined dysregulation of IL-18 bioactivity.

Cytotoxic dysfunction of NK cells has been reported as one of contributing features of MAS susceptibility in sJIA; however, there is some conflicting data (15, 23, 26, 29). Several studies demonstrate decreased NK cell cytotoxicity, but normal cytotoxicity was detected when the cytotoxicity was assessed relative to the decreased number of NK cells. The genetic variations in cytotoxic-related genes have been reported in some studies (24, 25); however, the genetic association with MAS development seems to be applied only to some patients (15). Accumulating evidence supports the notion that transient NK cell dysfunction rather than intrinsic cytotoxic defect is a primary feature of sJIA, which is substantiated by the reversible hyporesposiveness toward IL-18 and transient cytotoxic dysfunction due to NK lymphopenia and/or decreased expression of cytotoxic proteins (26–31). The origin of such abnormal phenotype of NK cells is still elusive, but is estimated to be caused by aberrant cytokine profile including IL-18 and IL-6 (29). The acquired NK cell dysfunction may contribute to decreased killing of activated T-cells and macrophages and sustained abnormal cytokine environment, which result in defective termination of the immune responses (29). A recent study using a sJIA mouse model supports a regulatory role of NK cells in the development of the disease *via* a NKG2D-dependent control of activated immune cells (120). More recently, in adult sHLH cohort, similar transient lymphopenia and decreased capacity of IFN-γ production in NK cells (i.e., exhaustive phenotype) but no major intrinsic defect in cytotoxicity-related genes were also observed (121). Such exhaustive phenotype or immunoparalysis seems to be rather common in hyperinflammatory syndrome including severe COVID-19 (122). The unresolved issue is whether this acquired phenotypical alteration offers pathological significance to MAS or sHLH occurence. Indeed, a recent investigation suggests that, rather than through cytotoxic dysfunction, excess IL-18 independently causes T-cell-mediated hyperinflammation (123). It is also unresolved whether the correction of such exhaustive phenotype can terminate inflammation.

From a diagnostic perspective, the levels of IL-18 can serve as a parameter for distinguishing MAS from fHLH or other SAIDs unrelated to MAS; furthermore, the IL-18/CXCL9 ratio slightly improves the diagnostic accuracy (16). A recent report illustrates a patient with XIAP deficiency who was efficiently diagnosed by evaluating the levels of serum IL-18 (124). In PAID, hyper-IL-18 is specifically linked with *PSTPIP1* pathogenic variants rather than with clinical phenotypes (59). Swift diagnosis and prompt initiation of appropriate therapy is absolutely imperative for achieving a favorable prognosis. Thus, the diagnostic utility of IL-18 is noteworthy in the MAS/HLH field.

Specific inhibition of IL-18 or IFN-γ exerts therapeutic effects on some IL-18-related SAIDs (36, 49, 53, 70, 74, 107), which indicates a pivotal role of IL-18 in certain inflammatory conditions. Whether these therapeutic effects rely on direct inhibition of proinflammatory features of these cytokines or correction of abnormal cytokine environment is still elusive. What is noteworthy is that the inflammatory phenotype of these disorders may not only rely on the levels of serum IL-18, but also on other humoral and/or cellular factors or the tissue specificity. Careful observations should be needed to discuss if these molecular-targeted therapies are generally effective for IL-18-related SAIDs.

The origin of chronic hyper-IL-18 is diversified and possibly depends on multifactorial pathways. Previous observations substantiate a close link with MAS/HLH predisposition; however, chronic elevation of serum IL-18 may not solely be a factor for susceptibility to MAS/HLH. From a clinical point of view, IL-18 and its related-molecules potentially serve as diagnostic markers and have been adopted as promising therapeutic targets. Therefore, recognizing IL-18-mediated SAIDs and unraveling the certain biology of IL-18 is significant.

## Author contributions

HM and TW wrote and critically revised the manuscript. All authors have approved the final manuscript. All authors contributed to the article and approved the submitted version.

## Conflict of interest

The authors declare that the research was conducted in the absence of any commercial or financial relationships that could be construed as a potential conflict of interest.

## Publisher’s note

All claims expressed in this article are solely those of the authors and do not necessarily represent those of their affiliated organizations, or those of the publisher, the editors and the reviewers. Any product that may be evaluated in this article, or claim that may be made by its manufacturer, is not guaranteed or endorsed by the publisher.
